# Children’s Diets and Planetary Health: A Study in Wroclaw, Poland, and Sydney, Australia

**DOI:** 10.3390/foods13223536

**Published:** 2024-11-06

**Authors:** Agnieszka Orkusz, Diana Bogueva

**Affiliations:** 1 Department of Biotechnology and Food Analysis, Wroclaw University of Economics and Business, 53-345 Wroclaw, Poland; 2 Curtin University Sustainability Policy Institute, Curtin University, Perth 6102, Australia; diana.bogueva@curtin.edu.au

**Keywords:** child, diet, kindergarten, menus, nutrition, planetary diet, social marketing intervention

## Abstract

Meals served to children should not only satisfy hunger and taste preferences but also be nutritionally adequate. Nutrition in early childhood is critical, as children spend a significant portion of their day in kindergarten or preschool, making these settings key contributors to their overall dietary intake. With the rising prevalence of nutrition-related health conditions among children, early interventions are essential for developing and establishing lifelong healthy eating habits. This study assessed the nutritional value and quality of children’s diets in two distinct settings: kindergartens in Wroclaw, Poland, and preschools in Sydney, Australia, evaluating their alignment with the planetary health diet. The research analysed 10-day menu cycles from five kindergartens in Wroclaw and the contents of lunchboxes from five preschools in Sydney’s Upper North Shore area. A total of 100 menus were reviewed in Poland, while 100 children’s lunchboxes were assessed in Australia. Different analytical methods were employed: the Diet 6D software program for the Polish menus and the Food Consumption Score for the Australian lunchboxes. Both methods revealed dietary imbalances, such as excessive intake of protein, vitamin A, salt, and sugar, alongside deficiencies in calcium, vitamin C, and vitamin D. The study concluded that children’s diets should adhere to nutritional guidelines, meeting both Polish and Australian standards, and align with the principles of the planetary health diet. To achieve this, nutritional education is essential for kindergarten staff in Poland, while targeted educational interventions are needed for parents and children in both Poland and Australia, promoting health and environmental sustainability through better nutrition.

## 1. Introduction

Adequate childhood nutrition is crucial for future health, supporting growth, proper bodily function, and a strong immune system while helping to prevent obesity and reduce the risks of chronic diseases from an early age [1]. Health-related habits, including regular exercise and good nutrition, developed during childhood offer lasting benefits and significantly contribute to overall health in adulthood [2,3]. Therefore, meals served to children not only satisfy hunger and taste preferences but also provide high nutritional value, exposing them to a variety of food products from different food groups and fostering sustainable dietary patterns. As children grow, these patterns can promote optimal health and reduce environmental impact [4].

While kindergarten nutrition does not cover the entire day, it plays an important role in fulfilling a significant portion of children’s daily dietary needs. Given that children typically spend 7–8 h a day in kindergarten [5,6], it is expected that preschool nutrition should provide approximately 75% of a child’s daily energy and nutritional requirements [6,7]. Ensuring proper nutrition during this time is essential for children’s health and helps mitigate the risk of health issues later in life. The nutrition provided should adhere to established dietary recommendations, meeting the necessary standards to ensure all necessary nutrients are included [8]. Early-childhood nutritional interventions, such as school meal programs and hands-on approaches (e.g., cooking classes or food education), have been shown to lead to significant improvements in cognitive, behavioural, health, and academic outcomes [9].

In Poland, nutritional standards are guided by several benchmarks including the Estimated Average Requirement (EAR), which meets the needs of 50% of the population, and the Recommended Dietary Allowance (RDA), which covers nearly all individuals. When data are insufficient to establish the EAR or RDA, Adequate Intake (AI) is used. AI is determined based on the average nutrient intake observed in a specific population group [8]. The Upper Safe Level (USL) indicates the maximum intake level of a nutrient unlikely to cause adverse health effects. For children, who are particularly vulnerable to nutrient excesses, meals should be planned according to the RDA (or AI where the RDA is unavailable) [10] to ensure the nutritional needs of nearly all children in the group are met. Energy requirements are generally determined by the EAR, reflecting average energy needs regardless of age [10].

According to the Australian dietary guidelines, children should consume a diverse range of foods from all five food groups: different types and colours of vegetables, fruits, grains, proteins, and dairy, while minimizing the intake of foods high in saturated fat, added salt, and sugar, and focusing on drinking plenty of water and avoiding sugary and fizzy drinks [11]. As primary food providers, parents play a crucial role in selecting nutritious foods and preparing healthy meals for their children’s lunchboxes. By encouraging regular consumption of these healthy choices, parents help establish the foundation for lifelong healthy eating habits. Research also highlights that caregiver involvement in supporting and modelling healthy food behaviours is essential for fostering these habits in children [12].

### 1.1. Planetary Health Diet

**Figure 1:**
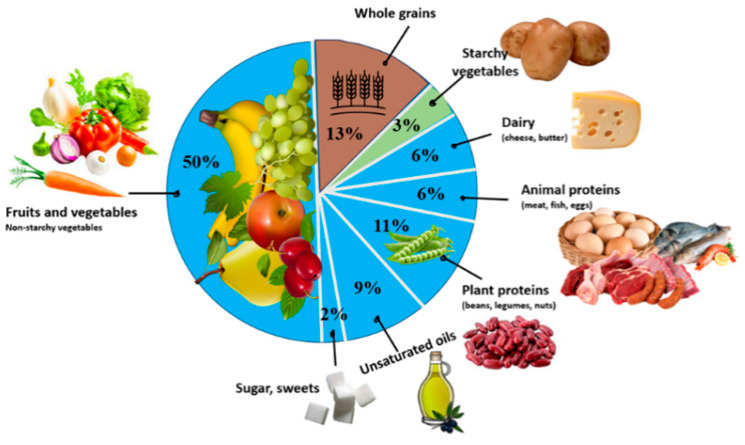
EAT–Lancet planetary health diet, adapted from the EAT–Lancet Report, Willett at al., 2019 [13].

In 2019, the EAT–Lancet Commission, a consortium of experts from 16 countries, released a landmark report introducing the concept of “planetary health” and the “planetary health diet”, also known as the EAT–Lancet diet (see Figure 1). This report emphasized the critical role that diets play in both human health and environmental sustainability, advocating for a unified global approach to transforming food systems. The planetary health diet not only links dietary patterns to human health and the environmental sustainability of the planet but also provides guidelines for daily consumption ranges across different food groups. This diet aims to optimize human health while considering environmental impact [13]. It is characterized by a high intake of plant-based foods—such as whole grains, fruits, vegetables, nuts, and legumes—paired with low amounts of animal products and refined grains. The diet is designed to be flexible, accommodating local and individual circumstances, traditions, and dietary preferences [14].

The EAT–Lancet planetary health diet has sparked important discussions around the feasibility of achieving equitable dietary transformation across diverse populations and food system contexts [11,15]. It also opens opportunities for international and national commitments to policy measures and actions aimed at making healthy and sustainable foods more available, accessible, and affordable. A diet rich in plant-based foods and lower in animal products offers both improved health and environmental benefits [13]. For those involved in preschool nutrition, the adoption of a planetary diet is particularly important [10]. Kindergarten staff should educate children on what constitutes a “healthy” plate, emphasizing that diet is a crucial component of good health. Additionally, it has become increasingly important to teach children about the environment and climate change impacts of their food choices.

### 1.2. Meeting Nutritional Requirements

Poor diets in early childhood can lead to deficiencies in essential vitamins and nutrients, weakening children’s immune systems. For instance, a diet lacking in vitamin A can impair vison and increase the risk of blindness [16]. Ensuring that children meet their nutrient needs early in life can be challenging, especially as they may be unfamiliar with the taste and benefits of many nutritious foods. However, in countries like Poland and Australia, where parents generally have access to a variety of age-appropriate foods, it should not be difficult to provide proper nutrition for their children.

Unfortunately, nutrition in Polish kindergartens often falls short of meeting these nutritional requirements, with many institutions not adhering to proper dietary guidelines [6,17].

Attention is primarily drawn to the excessive intake of protein, vitamin A, and salt, along with deficiencies in calcium and vitamin D [6,18]. Additional irregularities include a lack of vegetables and fruit in meals, excessive use of sugar and sweeteners in beverages, infrequent inclusion of fish, and an over-reliance on fried dishes throughout the week [6,10].

In Australia, food provision is not centralized, and kindergartens do not provide meals as they do in Poland. The nutritional quality of food is similarly impacted because the responsibility largely falls on families to supply food from home. This leads to significant variation in food quality across preschools and early-childhood centres. The meals often fail to meet national dietary recommendations for both quality and quantity, adversely affecting the well-being of toddlers and preschoolers [19]. Common issues include insufficient and inappropriate food, often consisting of low-cost, high-carbohydrate options with minimal protein and high levels of saturated fat [19]. This inadequate nutrition not only fails to support proper brain function and overall health but can also negatively impact children’s behaviour throughout the day.

This study aimed to assess children’s diets in five state kindergartens (A, B, C, D, and E) in the Wroclaw district, Poland, and five private preschools (F, G, H, I, and J) in the Upper North Shore area of Sydney, Australia. By observing and analysing 10-day menus, the research sought to compare the meals served in these institutions and evaluate their compliance with planetary health diet nutritional requirements. The study focused on three key areas using different assessment methods: (1) estimating the energy and nutritional value of the diets in the selected kindergartens in Poland; (2) assessing the frequency and intake of specific foods and nutrients in the preschools in Australia; and (3) evaluating the incorporation of planetary health diet principles into the meals provided in both countries to identify whether there are similar nutritional challenges shared between them.

## 2. Materials and Methods

### 2.1. Data

Data were collected in 2022 in Poland and in May 2023 in Australia. The methods of collection are described below.

#### 2.1.1. Polish Segment

This study evaluated menus from five randomly selected state preschools (A, B, C, D, and E) located in the Wroclaw district, Poland. In each kindergarten, children aged 4–6 were divided into 5 and 7 groups, with each group consisting of approximately 25 children. The selection process was based on a lottery-style random draw. This method involved first assigning a unique number to each state preschool within the dataset. Then, a predetermined number of preschools were selected by randomly drawing numbers from the pool. This approach ensured that every preschool in the dataset had an equal chance of being chosen, thereby reducing bias and making the selection process fair and objective for the study. In this way, all preschools, regardless of their characteristics, had the same probability of being included. The total, 806 children aged 4–6 years, who benefited from preschool nutrition, were included in this study. The selected kindergartens in the Wroclaw district offered three daily meals: breakfast, lunch, and afternoon tea. The menus were analysed over a ten-day period, detailing the specific products used and their weights. The analysis excludes part-time facilities due to fewer meals being offered, as well as preschools attended by younger children and those that relied on external catering services.

All kindergartens had on-site kitchens where meals were prepared. The study evaluated 10-day menus from both the winter and spring seasons in each of the five Wroclaw kindergartens. In total, 100 menus were analysed, with 50 menus from each season. Portion sizes were estimated based on the quantity of products, meals, and drinks specified in grams on the menus. For the purpose of the analysis, it was assumed that children consumed all of the meals served.

The Diet 6D software [20] was used to calculate the energy value and nutrient content of the meals. This analysis included protein, fat, carbohydrates (including glucose, fructose, sucrose, lactose, and starch), fibre, vitamins (thiamine (B_1_), riboflavin (B_2_), niacin (B_3_), pyridoxine (B_6_), and vitamins A, C, D, and E), minerals (sodium (Na), potassium (K), calcium (Ca), phosphorus (P), magnesium (Mg), iron (Fe), zinc (Zn), copper (Cu), and manganese (Mn)), cholesterol, fatty acids (saturated, monounsaturated, and polyunsaturated), and salt. The results were then compared with the applicable standards and recommendations for children aged 4–6 years [8].

The energy standard was based on the Estimated Energy Requirement (EER), with a daily energy intake of 1400 kcal assumed according to established guidelines [8]. Reference Intake ranges for fat and carbohydrates were expressed as a percentage of total energy requirements. Standards for protein, selected minerals (calcium, phosphorus, magnesium, iron, zinc, and copper), and vitamins (thiamine, riboflavin, niacin, pyridoxine, and vitamins A and C) were aligned with the Recommended Dietary Allowance (RDA). For other nutrients, such as sodium, potassium, manganese, vitamins D and E, fibre, and polyunsaturated fatty acids (PUFA n-3, PUFA n-6), Adequate Intake (AI) standards were applied.

The norm for preschool nutrition was set at 75% of the daily recommended energy and nutrient intake for children aged 4–6. Total preschool consumption values that deviated by ±10% from the standard were considered acceptable. The content of vitamins (A and B_6_) and minerals (Mg, Zn, and Cu) was also compared to 75% of the tolerable upper intake level (UL) as proposed by the European Food Safety Authority [21].

Additionally, each facility was evaluated on the implementation of planetary health diet principles, including the emphasis on wholegrain cereals, legumes, and nuts, and the restriction of meat in the meals provided.

#### 2.1.2. Australian Segment

For the Australian segment of the study, data were collected from five preschool kindergartens (F, G, H, J, and K) located in the Upper North Shore area of Sydney, New South Wales (NSW). The Australian preschools were chosen using a lottery-style random draw, similar to the Polish preschool selection, ensuring that each preschool had an equal probability of being included in the sample. This study marks the first assessment of preschool nutrition in these preschool institutions (caring for children from 8 a.m. to 3 p.m.). In line with the Australian government-approved education system, preschool kindergartens play a key role in preparing children for school.

For children aged 4–8 years, the Australian guidelines recommend a daily intake of 1½ servings of fruit, 4½ servings of vegetables, 1½ to 2 servings of dairy, 4 servings of grains, and 1½ servings of lean meats, eggs, nuts, seeds, or legumes [11]. This observational study aimed to measure the frequency of food and beverage consumption in the preschool setting, with a focus on lunches and morning tea snacks. Over a 10-day period, the contents of 100 lunchboxes were observed during morning tea and lunch times across five randomly selected preschool groups—one group from each selected kindergarten. Each group comprised 20 children aged 3–5 years. The research was conducted solely through distant observations of the children’s lunchbox contents, with no direct interaction with the children.

During the observation, information was recorded about the food content of each child’s lunchbox menu during morning tea and lunch. Unlike in Poland, where kindergartens provide meals through a centralized system, in Australia, the system requires parents to prepare and pack their child’s food at home to be consumed at designated times of the day, namely morning tea and lunch. Despite the lack of centralized food preparation, the New South Wales Education guidelines recommend providing healthy food that children will enjoy and eat, with an emphasis on promoting healthy eating habits [22]. Additionally, preschool management often provides food recommendations, primarily focused on food allergies. For instance, parents are advised not to pack nuts in lunchboxes to prevent accidental food sharing between their child and another child with a nut allergy, thus avoiding potential adverse health reactions.

### 2.2. Statistical Analysis

Two different methods were used for the quantitative data collection and analysis, aiming to identify dietary patterns and evaluate their compliance with planetary health diet nutritional requirements.

#### 2.2.1. Polish Segment

The quantitative analysis of the menus was conducted using the Dieta 6 software program. A two-way analysis of variance (ANOVA) was employed to assess variations in the energy value and nutrients content of the Polish preschool menus. This analysis considered factors such as the specific kindergarten, season (winter or spring) and interactions between these factors. When a significant main effect or interaction was detected, the mean values were further examined using Tukey’s multiple range test to determine specific differences. The results were presented as the mean ± standard deviation. The experimental data were analysed using Statistica version 13.3 [23], with differences considered significant at a probability level < 0.05.

#### 2.2.2. Australian Segment

A quantitative Food Frequency Questionnaire (FFQ) was used to record and assess the food consumption frequency of Australian preschoolers [24]. The FFQ method provided data on the usual intake of specific foods and nutrients consumed by the preschoolers, offering a more valid indicator of the relationship between their dietary patterns and health outcomes. Unlike the Polish segment of the study, this method did not aim to capture the energy values and nutrient content of the foods consumed. It was simply intended to capture whether the foods consumed are in line with the planetary health diet nutritional requirements.

### 2.3. Ethical Statement

Ethical review and approval were waived for this study in Poland as the research was based on an evaluation of the preschool menus based on the computer program Diet 6D. The Australian part of the study was covered by the ethical approval of the research obtained in Australia by the Curtin University Human Research Ethics Committee (approval number: HRE2022-0041), in accordance with the National Statement on Ethical Conduct in Human Research, 2007.

## 3. Results

### 3.1. Polish Kindergartens

The analysis of variance showed that manganese, vitamin B2, niacin, vitamin C, vitamin E, glucose, sucrose, fructose, lactose, fibre, PUFA n-3, PUFA-n-6, and the PUFA n-6/n-3 ratio were significantly affected by kindergarten type (*p* < 0.05). The seasonal factor was not significant for any nutrients, and a two-way interaction was observed only for glucose and fructose (Table 1). The energy value, protein, fat, and carbohydrate content of the meals did not differ significantly by preschool and season (Table 1). However, kindergarten D had excessive energy intake in winter (Table 2), exceeding 75% of Estimated Energy Requirement (EER), and protein levels across all institutions were double the Recommended Dietary Allowance (RDA) (Table 2).

In winter, the fat content of meals served in kindergarten E met recommendations, while other kindergartens had fat levels below 75% of the recommended intake (RI). Fat is a source of fatty acids, including essential polyunsaturated fatty acids that must be supplied with food. Despite this, the children’s diets lacked sufficient n-6 and n-3 polyunsaturated fatty acids (PUFAs). Additionally, saturated fatty acid levels were too high in kindergarten D during winter and spring, and in kindergarten E during winter (Table 2). Except for kindergarten D, carbohydrate levels were adequate across all facilities (Table 2). Full-time kindergarteners (7–8 h per day) should limit sucrose intake to a maximum of 26.25 g, yet all kindergartens exceeded this recommendation (Table 2). Sucrose, primarily found in processed foods and used as refined sugar at home, contributed to this excess. Dietary fibre levels were above the recommended levels in every kindergarten (Table 2).

Mineral content in the children’s diets showed no significant differences based on kindergarten or season, except for manganese. Each preschool exhibited irregularities in mineral content, with calcium intake falling below 75% of the RDA and excess sodium, potassium, phosphorus, magnesium, zinc, copper, and manganese levels exceeding 75% of the RDA (Table 2). Sodium, potassium, and copper levels surpassed the standards by more than double in all kindergartens (Table 2). Importantly, the tolerable upper intake levels (ULs) for magnesium, zinc, and copper were not exceeded (Table 2). Iron content was consistently below 75% of the RDA in all kindergartens, except institution D in winter, ranging from 5.57 mg to 7.81 mg (recommended standard: 7.5 mg) (Table 1). Manganese levels ranged from 1.90 g to 3.58 g in winter (Table 2).

Riboflavin, niacin, vitamin C, and vitamin E content varied significantly across preschool institutions (Table 2). The analysed menus met children’s vitamin needs to different extents. All preschool institutions showed excesses of vitamins B1, B2, B6, C, and A (intakes above 75% of RDA) while also displaying a deficiency in vitamin D (intake below 75% of the Adequate Intake (AI)) (Table 2). Notably, vitamin A intake exceeded 75% of the tolerable upper intake levels in winter at kindergartens D and E, as well as in institutions A and D during spring. Niacin and vitamin E intake also varied by preschool institution (Table 2).

All kindergartens exhibited irregularities in salt content, with menu levels ranging from 4.06 g to 5.31 g (Table 1), exceeding the recommended intake by more than double (Table 2). The meals served to children comprised three components: breakfast, lunch, and afternoon tea, with appropriate intervals of 4 and 3 h between meals, respectively [9].

Feeding practices were similar across institutions. For breakfast, children typically received bread or wheat rolls with butter, accompanied by a high-protein item (such as eggs, sausages, or cheese) or a high-carbohydrate option (like jam or marmalade). Less frequently, they were served milk with cornflakes or flavoured cereals (e.g., chocolate). Breakfast was often complemented by fruit (usually an apple or banana) or vegetables (tomato or carrot). Beverages primarily included tea, caffeine-free coffee, and occasionally cocoa, all prepared fresh and sweetened with sugar.

The observed lunch patterns in Poland reflect a thoughtful balance of nutritional adequacy, cultural heritage, and sensory appeal. Lunch typically included soup and a main course. The most commonly served soups were chicken broth, tomato, potato, and barley, all made from natural ingredients and vegetable or meat stocks. These soups provide hydration and essential nutrients, reflecting Polish culinary traditions that prioritize locally available ingredients, such as potatoes [25]—a typical staple that offers complex carbohydrates, key nutrients like vitamin C, potassium, and dietary fibre [26]. The soups not only offer protein and vitamins but also contribute to a comforting meal experience. This is also in line with the EAT–Lancet reference diet recommendation for consumption of tubers or starchy vegetables (50 g/day), specifically potatoes and cassava [15]. The main course usually consisted of potatoes (with rice or pasta less frequently) paired with meat and cooked or raw vegetables. Furthermore, the preference for freshly prepared dishes over processed options indicates a growing awareness of health, reflecting contemporary dietary trends that emphasize whole foods and minimize additives. Sweet dishes were less common but included pancakes with cottage cheese and sugar, rice with strawberry sauce, and sweet dumplings. Freshly prepared compote was generally served with processed fruit and vegetable drinks and was offered less often. While sweet dishes like pancakes and rice with strawberry sauce are less common, their presence adds a touch of indulgence, highlighting the importance of dessert in Polish culture. Overall, this approach to lunch underscores the role of traditional foods in promoting well-being and fostering cultural identity.

For afternoon tea, children were usually given sweet rolls, jellies, bread with chocolate spread, and occasionally pudding (chocolate or vanilla). Sandwiches with cold cuts, including smoked meat, pâté, egg, or jam were served from time to time. Warm tea sweetened with sugar, milk, or sweet milk drinks were provided, while fruit was rarely offered, except for canned fruit like peaches.

Meals were prepared using various techniques, including boiling, stewing, frying, and baking. However, the analysed menus lacked seasonal produce, whole grains, legumes, fish, and high-sugar-containing products, e.g., jams, preserves, fruit in syrup, sweet bread, breakfast cereals, and sweet drinks. Preschoolers also did not have consistent access to drinking water, and herbs and spices were minimally used, with only pepper, paprika, and caraway being common.

Additionally, none of the preschool institutions adhered to the principles of the planetary diet, as evidenced by the menus (Table 3).

The children’s diet lacks a variety of high-quality plant-based foods, featuring low levels of whole grains, legumes, and nuts. In contrast, meals contained a high proportion of animal products, primarily meat and eggs (Table 3). Meals were served on plates rather than being offered as a self-selection buffet, limiting children’s ability to choose items such as vegetables and fruit or the presentation of dishes (e.g., meat with or without sauce).

### 3.2. Australian Preschool Kindergartens

The Food Frequency Questionnaire (FFQ) was adapted to reflect the culturally specific Australian food context and used as a dietary assessment instrument to capture the usual food consumption of selected Australian preschoolers based on a predefined list of food groups [24].

The Australian part of the study aimed to observe dietary diversity and food frequency, assessing the nutritional importance of various food items for parents and preschoolers. Food items were grouped into eight (n = 8) standard food groups, and their frequency of consumption was recorded daily over a 10-day period (two weeks from Monday to Friday) to evaluate the maximum intake. While information on consumption frequency was collected, details on food preparation methods and meal types were limited. Table 4 outlines the frequency of consumption for different food items by preschool group. Across all five preschool groups (F, G, H, I, and J, each with 20 children), a total of 6490 instances of food consumption from the eight categories were recorded.

**Figure 2:**
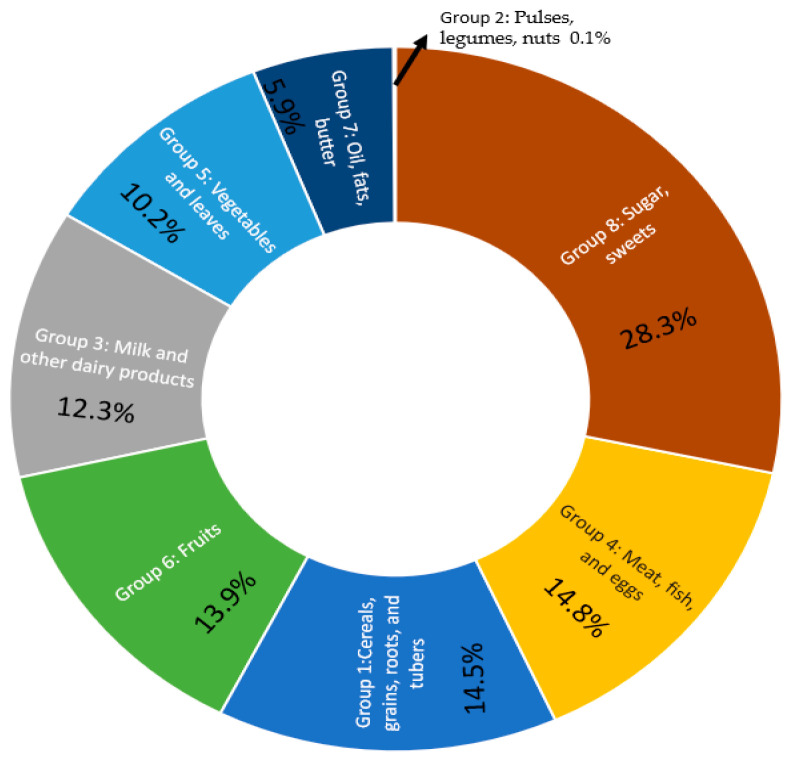
Consumption frequency by food group (as a percentage).

Among all food groups, Group 8 (sugar and sweets) was the most frequently consumed, with a total of 1840 instances (28.3%) recorded over 10 days. Children regularly consumed various foods, drinks, and snacks containing added sugar with consumption nearly equal during morning tea and lunch. In contrast, other food groups were primarily consumed during lunch break. These findings align with previous results from the Australian Health Survey, which assessed food and nutrient consumption in children aged 2 years and older [27]. Preschool G stood out for its relatively lower consumption of sugar and sweets. Conversely, Group 2 (pulses, legumes, and nuts) was the least preferred, with only 10 instances (0.1%) recorded across the selected preschools. The highest consumption of this food group was noted in preschool G, primarily among children from multicultural backgrounds who spoke English as a second language. Foods from Group 4 (meat, fish, and eggs) accounted for 14.8% of consumption, followed closely by Group 1 (cereals, grains, roots, and tubers) at 14.5%. Figure 2 illustrates the total frequency percentage of each food group consumed in preschool. The FFQ aids in interpreting dietary habits and determining appropriate consumption limits for various food groups. Lunchbox observations revealed that children attending preschool G had notably different dietary choices compared to the other preschools included in the study (see Table 4). Children in G preferred fruits and vegetables (Groups 5 and 6), consuming them far more frequently than children in other preschools. This trend was also observed with milk and cheese (Group 3) and meat (Group 4). Furthermore, an interesting behaviour was noted: all children at preschool G constantly began both morning tea and lunch by eating the fruits and vegetables from their lunchboxes first before moving on to the rest of their meals, prepared by their families or themselves.

Table 5 compares the recommendations of the EAT–Lancet planetary health diet with the contents of Australian preschoolers’ lunchboxes, ranked from the highest to lowest consumption frequency of each food group observed. The EAT–Lancet planetary health diet prioritized fruits and vegetables, followed by whole grains, plant proteins (e.g., beans, legumes, and nuts), unsaturated plant oils (e.g., olive oil), animal proteins (e.g., beef, lamb, pork, chicken, fish, and eggs), dairy, starchy vegetables, and sugar and sweets (see Figure 1).

## 4. Discussion

The statistical analysis of the results from the five state preschools (A, B, C, D, and E) in the Wroclaw district, Poland, showed no significant differences in energy value or the content of essential nutrients (protein, fat, and carbohydrates) across institutions or season (see Table 1). This consistency suggests that Polish preschools adhere to standardized meal-planning practices, likely due to national regulations governing preschool nutrition.

However, the energy content of meals, while meeting the recommended guidelines, contradicts previous studies that highlight an excessive energy intake in similar settings [6,18,28]. One explanation for this could be the increasing awareness and stricter enforcement of nutritional standards in recent years. Moreover, facility D’s variation in butter use between seasons suggests a seasonal influence on meal composition, which might reflect availability and cultural food preferences. In contrast, the Australian study revealed that butter is used sparingly among preschoolers, primarily as a sandwich spread to hold other ingredients together (see Table 4).

In the Australian study, preschool G’s closer alignment with the EAT–Lancet planetary health diet demonstrates the growing influence of global health guidelines in shaping early-childhood nutrition. The differences observed between preschool G and the other preschools likely reflect the socioeconomic or cultural variations within the communities. Parents in preschool G may be more attuned to global trends in sustainable eating, potentially due to greater access to information or stronger emphasis on health and environmental concerns. Cultural predispositions around eating practices also play a significant role, as food habits and preferences are often shaped by the values, attitudes, and norms transmitted across generations. This intergenerational transmission of food culture helps explain the relative stability of eating behaviours over time, contributing to the persistence of certain dietary patterns within specific cultural or social groups [29].

Regarding nutrient deficiencies, the lack of sufficient polyunsaturated fatty acid (PUFA) intake—considered healthy fats according to the EAT–Lancet diet [13]—in Polish preschool diets is concerning, particularly from the n-3 and n-6 families (see Table 2). PUFAs play a critical role in cognitive and neurological development. Previous research [28,29,30,31,32] has extensively documented the long-term risks of PUFA deficiency, including potential impacts on brain function and the immune system. As essential nutrients that must be obtained through food, PUFAs are vital for brain function, heart health, and cell growth due to their anti-inflammatory properties [30,31]. A deficiency of n-3 EFAs can impair brain development, with 90% of early brain development occurring by age of 5–6, although some evidence extends this window to age 6–8 [32,33]. Docosahexaenoic acid (DHA, 22:6 n-3) is particularly important for eye health, including retinal maturation, visual acuity, and mental development. PUFAs also play a key role in neurotransmitter synthesis and immune system function. An imbalanced ratio of n-6 to n-3 fatty acids can negatively impact lipid profiles, increase oxidative stress, and heighten the risk of obesity [34]. The lack of PUFA sources, especially DHA, in the Polish menus indicates a gap that could be addressed through better menu planning, as fish and plant-based sources are often underutilized in institutional settings. Another study reported that fish was not among students’ favourite food choices, with many avoiding school meals on days when it was served. However, by offering more appealing options like fish cutlets, meatballs, and baked fish with vegetables, schools were able to increase student consumption [35]. This deficiency contrasts with Australian preschools, where lunchboxes often contain fish and other PUFA-rich foods, likely due to parents controlling their children’s food choices and providing greater dietary variety. However, other studies indicates that later, when in primary school, Australian children’s diets shift toward a high intake of discretionary foods—energy-dense, nutrient-poor items high in saturated fat, sugars, and sodium—which contribute over a third (38.5%) of their daily energy intake, while they fall short of national recommendations for vegetables, fruits, and dairy [36]. Adequate protein and essential amino acids are crucial for normal child growth and development. However, monitoring protein intake is essential due to its varying effects across different life stages. Garcia-Iborra et al. (2023) highlight that while increased protein consumption may benefit older adults, it is discouraged in infancy due to potential links to obesity later in life [37]. Notably, research on the long-term consequences of elevated protein intake in children and adolescents (ages 4–18) is limited, despite evidence indicating that their intake often exceeds recommended levels by two to three times in developed countries [37]. The evaluation of Polish kindergarten menus has revealed that protein content frequently exceeded the RDA by more than double, largely due to high meat and processed meat product consumption (Table 3). Similarly, findings from the FFQ observation in Australian preschools showed that most children consumed enough animal-based products, fish, and dairy daily (see Table 4). Common food items included ham, chicken, canned tuna, salami, eggs, mince pies, meatballs, spaghetti Bolognese, fish fingers, fruit yogurt, milk, and cheese. Children’s eating habits are shaped through exposure to different foods, tastes, and textures, as well as by observing their surrounding environments and the consumption behaviour of others, their families, and peers [38]. In this context, the content of Australian preschoolers’ lunchboxes is significantly influenced by parents, caregivers, and stronger peer dynamics. Given that children at this age have limited autonomy in making food choices, their meals tend to be high in proteins, alongside popular sugary foods and drinks, raising concerns about the potential implications for their overall health and development.

Excessive protein intake in children’s diets is a common concern [6,39], with Australian dietary guidelines recommending 20 g of protein per day for children aged 4–8 [40], while United States dietary guidelines suggest that protein should constitute 10% to 30% of a child’s total energy intake, with the rest from carbohydrates and fats [41]. A healthy diet should also include foods rich in calcium and iron to support muscle and bone growth [42].

Although Polish standards have yet to define an upper limit for protein intake, efforts are being made to establish one [8]. High protein intake from an early age may lead to increased energy intake, fat mass, and potential long-term health issues like overweight and obesity, which is particularly concerning given the rising childhood obesity rates, especially among preschoolers [43]. Excess protein is metabolized for energy [8], but there is no evidence linking high protein intake to bone mass issues. Still, it is advisable to avoid very-high-protein diets (i.e., over 2.0 g/kg body weight/day) if calcium intake is low (i.e., less than 600 mg/day) [44].

Carbohydrate content in the analysed Polish menus, except for kindergarten D, was appropriate (Table 2). However, an excess of sucrose, mainly from processed foods and refined sugar used in households, was found across all facilities. Excessive sucrose and simple sugar intake is linked with increased risks of obesity, insulin resistance, diabetes, metabolic syndrome, tooth decay, and cancer [45,46].

Sucrose in the analysed preschool menus mainly came from refined sugar added to drinks (tea and fruit compote), sweet dishes (e.g., pancakes with cottage cheese), and processed products (breakfast cereals, cakes, cookies, sweet drinks, fruit yoghurts, and jams). A similar trend was observed in the majority of Australian preschoolers’ lunchboxes (F, H, I, and J), exceeding both Australian dietary guidelines [40] and the EAT–Lancet planetary health diet [13]. To reduce sugar intake in Polish kindergartens, healthier alternatives can be introduced: offer water and unsweetened drinks infused with fruit or mint and replace sugary drinks with freshly squeezed fruit and vegetable juices. Sweet snacks like cakes can be replaced with fresh or dried fruits, served raw or added to dishes, like fruit salads, mousses, and jellies for added sweetness and nutrition. Meals like pancakes can be served with fresh fruit and natural yogurt and with cheese or cottage cheese. For breakfast, natural oats, rye, and barley are healthier than sugary cereals. Similar recommendations apply to Australian parents, who can prepare healthier lunchboxes by opting for more plant-based food options and involving their children in healthier food choices.

The identified irregularities highlight the need for targeted educational interventions, such as social marketing strategies, to help educators promote healthy dietary choices [47]. A successful example is seen in Australian preschool G, where teachers positively influenced children’s prioritization of fruit and vegetable consumption during the observation period.

Implementing nutritional education for kindergarten staff, similar to that in Australian preschool G, can help prevent long-term consequences, as children may carry nutritional habits learned in kindergarten into adulthood. Since childhood is critical time for shaping positive behaviours, it is vital for staff to prepare meals following proper nutrition guidelines and impart reliable knowledge, fostering lifelong healthy eating habits.

The tested meals met children’s mineral needs to varying degrees. Across all kindergartens, there was a calcium deficiency and an excess of sodium, potassium, phosphorus, magnesium, zinc, and copper.

The research showed that the calcium content in children’s diets across all kindergartens was below the RDA standard (Table 2). However, the menu did not lack dairy products, aligning with planetary diet guidelines (Table 3). To ensure balanced nutrition, meal planning should prioritize not only the inclusion of all food groups but also the selection of nutrient-dense options. For kindergarten children, at least one portion of dairy products should be a fermented milk drink (e.g., yoghurts, kefir, buttermilk, or curds) or included as part of a meal. Calcium deficiencies can lead to increased excitability, neurological disorders, and elevated blood pressure. Adequate calcium intake is crucial for preventing diseases such as obesity, type 2 diabetes, and certain cancers (including breast, prostate, and colorectal cancer) [48,49,50].

Our research results align with the literature, confirming that calcium deficiency in children’s diets is a widespread issue [5,6]. Additionally, the analysis revealed that, across all kindergartens, the intake of sodium, potassium, manganese, phosphorus, zinc, copper, and magnesium exceeded Polish nutritional standards (with Na, K, and Mn intake higher than 75% of AI, and P, Zn, Cu, and Mg intake higher than 75% of the RDA). However, it is important to note that the ULs for magnesium, zinc, and copper, as set by the European Food Safety Authority, were not surpassed (Table 2). A balanced diet is essential, as children’s developing bodies are particularly vulnerable to nutrient imbalances.

Excessive salt intake is a common issue in children’s diets [6]. Adults often prepare meals based on their own taste preferences, overlooking the fact that children require significantly less salt. Early childhood and adolescence are crucial periods for establishing healthy eating habits, including appropriate salt consumption, which can impact long-term health. To reduce salt intake, low-sodium foods should be prioritized, and processed products like smoked meats, cold cuts, canned vegetables, and ripened cheeses should be limited. Additionally, salt should be minimized during food preparation, such as by avoiding salting potatoes, rice, and pasta, which are often served with already-seasoned items like meat or fish. Herbs and spices can serve as healthier substitutes for salt. Similarly, Australian preschoolers’ lunchboxes also contained high-sodium foods such as ham, salami, and meat pies [51].

Iron deficiency was evident in all Polish preschool meals, except for kindergarten C’s winter menu, where iron content met 75% of the RDA. This aligns with the literature, which also reports low iron intake in children’s diets [6,26]. Key iron-rich foods include parsley, dried legumes, lean meats, eggs, and wholegrain bread [20]. In the Australian preschoolers’ lunchboxes, iron-rich foods such as broccoli, leafy greens, vegetables, eggs, fish, chicken, and some legumes were observed. Iron deficiency can lead to anaemia with symptoms like pale skin, headaches, tongue inflammation, rough skin, and brittle hair and nails [52]. Furthermore, insufficient iron intake may increase the risk of depression [53,54].

In line with the EAT–Lancet planetary health diet guidelines, supplementing children’s diets with wholegrain cereals and legume-based dishes, while limiting meat and meat products, would be highly beneficial. This shift would not only provide essential nutrients, such as fibre, plant proteins, and vitamins, but also promote long-term health and sustainability. By incorporating more plant-based foods and limiting meat, children’s diets would align better with both nutritional needs and environmental goals, fostering healthier eating habits for the future.

The vitamin status of children’s diets is influenced by various factors and plays a crucial role in preventing and treating many diseases. Research indicates that even in resource-rich societies like Europe, achieving adequate vitamin intake can be challenging [55]. Fortunately, the reviewed Polish kindergarten menus demonstrated a positive vitamin profile, with high levels of vitamins A, B1, B2, B6, and C, all exceeding 75% of the RDA. However, vitamin D deficiency was noted (Table 2). While the UL for vitamins B1, B2, and C has yet to be established, and the UL for vitamin B6 was not exceeded; the intake of these appears excessive, likely due to high consumption of meat (particularly pork and offal) and eggs.

Other micronutrients such as thiamine and riboflavin are essential for supporting children’s bodily functions and helping to prevent developmental abnormalities. Currently, no negative symptoms have been reported from excessive consumption of these vitamins, as the body limits their absorption and increases urinary excretion [56]. Similarly, while excess pyridoxine (vitamin B6) is excreted in urine, long-term intake of doses exceeding 100 mg/day may lead to mild neurological symptoms [57].

The high vitamin C content in the examined preschool meals is attributed to the substantial inclusion of fruits and vegetables in Polish kindergartens’ menus, including processed drinks enriched with vitamin C. However, it is important to note that these beverages are often packaged in plastic, which can contain bisphenol A (BPA). This chemical can leach into food, leading to negative health effects. Research demonstrates that BPA generates reactive oxygen species, causing oxidative damage to the liver, kidneys, and testes in animal studies, and vitamin C can exacerbate this damage [58]. Therefore, it is advisable to avoid consuming vitamin-C-enriched beverages from plastic bottles. During growth and development, children’s bodies are particularly vulnerable to harmful substances, making it crucial for their diets to consist of minimally processed foods that are prepared fresh whenever possible. Similarly, Australian preschoolers also consume vitamin C rich foods, such as fruits and vegetables (see Table 4) [59]. This was particularly noticeable in preschool G, where the intake of fruits and vegetables was notably high, including items like oranges, mandarins, kiwi, strawberries, broccoli, capsicums, and tomatoes (see Table 5). Regular consumption of vitamin-C-rich foods from an early age is essential for forming and repairing red blood cells, bones, and tissues, which helps minimize bruising from falls. Additionally, vitamin C plays a vital role in maintaining healthy gums and acts as a powerful antioxidant, aiding in the prevention, control, and combat of infections [60].

Another essential vitamin for children’s health and development is vitamin A, which is found in animal products and various fruits and vegetables containing carotenoids [61]. Alarmingly, the vitamin A content in the diets of children at all Polish facilities, except kindergarten B, exceeded the tolerable upper intake level (see Table 2). In the observation of Australian preschoolers’ lunchboxes, sources of vitamin A were well presented, including fish in the form of canned tuna and fish fingers, cheese, eggs, and carrots across all facilities (see Table 4). However, excessive vitamin A can be toxic and teratogenic [61]. In the Polish kindergartens, the primary sources of vitamin A were eggs, rennet cheese, and butter, aligning with previous studies that reported high levels of vitamin A in preschool meals [6,39].

Additionally, vitamin D, which is crucial for maintaining bone and muscle health but is often globally deficient, was found to be of insufficient intake in children’s diets. Vitamin D is challenging to obtain from food, except for fish, which was regularly included in Australian preschoolers’ lunchboxes. Most vitamin D is synthesized in the skin when exposed to sunlight. In contrast, the Polish children’s diets, regardless of the kindergarten, were characterized by low vitamin D content (see Table 2). This deficiency was primarily due to the infrequent inclusion of fish in the meals. Vitamin D is crucial for proper calcium and phosphate metabolism, playing a key role in the development of the skeletal system [62]. In contrast with Poland, in Australia, the government recommends a daily intake of 5 μg (micrograms) of vitamin D [63], despite the county’s ample opportunity for year-round sunlight skin exposure, which allows for natural vitamin D synthesis. This recommendation is necessary because individuals, particularly those with light or pale skin, face a higher risk of developing skin cancer due to increased sun exposure. As a result, extensive sun protection, such as covering the skin and regular use of sunscreen, is advised. Vitamin D deficiency in children and adults can decrease intestinal calcium absorption, temporarily reducing blood ionized calcium levels [64]. When vitamin D levels are low, calcium is released from the bones to maintain the proper levels of Ca^2+^ in the blood, leading to bone demineralization. Prolonged vitamin D deficiency (hypovitaminosis D) in children can result in decreased bone density, causing skeletal deformities, particularly in the lower limbs [65]. Ensuring adequate levels of vitamin D through diet is vital, with oily fish and eggs being primary sources. Additionally, vitamin D is synthesized in the human skin through exposure to UVB solar radiation. During the winter–spring season in Poland, sunlight exposure is insufficient for this process, necessitating dietary sources or supplementation. It is important to educate kindergarten staff on the significance of vitamin D and its essential role in maintaining health.

Meal planning to meet the full range of nutritional needs is challenging. A 10-day planning cycle often helps in achieving better balance, especially concerning vitamins and minerals [10]. This study found that none of the Polish kindergarten menus fully met the nutritional guidelines (RDA and AI standards) or the planetary health diet recommendations. While the fibre content was adequate, the menus lacked the variety recommended by the planetary diet, which emphasizes not only vegetables and fruits but also nuts, legumes, and whole grains. A diverse selection of foods from all basic groups in appropriate proportions (Table 3) is crucial for providing essential nutrients, as no single food contains all the necessary nutrients.

Collective nutrition in Polish kindergartens should adhere to established guidelines and expert-developed norms [8]. The Ministry of Health regulation from 26 July 2016, aims to enhance the nutritional quality of meals provided to children and young people in educational institutions [66]. This regulation focuses on limiting the consumption of foods high in added sugars, sweeteners, fat, and salt, thereby promoting healthier eating habits within mass catering services for children and young people [66]. According to the regulation, fruit and vegetables must be included in every meal, and fried dishes should be limited to twice a week. However, research by Myszkowska-Ryciak and Harton (2018) [2] involving 706 kindergartens throughout Poland revealed that many institutions do not fully comply with these guidelines. Only about half of the kindergartens served vegetables and fruits with every meal, 46% used table salt, and a quarter (¼) added sugar to meals. The authors highlighted the need for further support to ensure adherence to the rules, suggesting that the current regulations are too general, allowing too much flexibility in meal planning. This underscores the importance of nutritional education and more specific dietary recommendations for kindergartens. Our research aligns with these findings, showing similar issues with high salt and sugar content in the sampled menus.

In the Australian part of the study, the results showed that parents generally prepared more nutritionally balanced lunchboxes for their children. However, excess sugar and sweets were consumed regularly in all participating preschools except for one (see Table 5). A combined parent–educator intervention could help promote healthier choices aligned with the EAT–Lancet planetary health diet and the Australian dietary guidelines [11]. Preschool G already exemplifies success in this direction, serving as a model for integrating these dietary recommendations into daily meals.

## 5. Conclusions

Regardless of the kindergarten, the analysed menus revealed significant nutritional inadequacy compared to dietary recommendations for preschool children, signalling the need for targeted modifications. None of the facilities fully implemented the principles of the EAT–Lancet planetary health diet. Notably, neither the Polish nor the Australian menus adequately limited meat consumption, incorporated seasonal and regional vegetables and fruits, or provided sufficient amounts of wholegrain cereal products and legume-based dishes. Regular reviews and adjustments are essential to better meet children’s nutritional needs, with modifications guided by ongoing research into energy and nutrient content to rectify any dietary imbalances.

A notable limitation of this study was the absence of data on the actual consumption by the children in both the Polish and Australian samples, along with a lack of precise information on salt used during meal preparation. In Australia, it remains important to investigate whether the lunchbox contents are predominantly influenced by parents or children, as this can inform the design of effective educational interventions, including social marketing initiatives. This is crucial for ensuring that children’s actual intake aligns with the recommended national dietary standards.

Both parts of the study shared the primary objective of assessing the nutritional value of preschool meals in light of the planetary health diet, although they used different methods due to contextual differences (Diet 6D software in Poland and the Food Consumption Score in Australia). Given these contextual differences, the study did not employ a standardized protocol but aimed instead to maintain a shared overarching goal of assessing nutritional alignment with the planetary health diet. Future research may benefit from establishing common objectives with a standardized protocol adaptable to different meal contexts to enable more direct comparisons between settings. Future studies may benefit from defining common objectives and a standardized protocol adaptable to each context for more direct comparisons. Despite these methodological distinctions, both locations aligned in their evaluation of dietary imbalances and informed interventions tailored to the specific needs of kindergarten staff in Poland and parents in Australia, with the shared goal of enhancing early-childhood nutrition.

To address these issues, comprehensive nutritional education is necessary for preschool staff, parents, and children in both Poland and Australia. These programs should emphasize the importance of nutrition in promoting health and highlight the connection between good dietary practices and environmental sustainability. By fostering awareness and responsibility, nutrition education can become a powerful tool for safeguarding and advancing both human health and the planet.

## Figures and Tables

**Table 1:** Mean values (± standard deviation) of energy value and nutrient content in the Polish preschool menus.

Energy	Season (S)	Kindergarten (K)					Significance Effects		
and Nutrients		A	B	C	D	E	K	S	K × S
Energy [kcal]	Winter	1031.88 ^ab^ ± 152.7	1041.28 ^ab^ ± 188.9	995.71 ^a^ ± 130.3	1159.86 ^b^ ± 159.8	1107.8 ^ab^ ± 112.3	ns	ns	ns
	Spring	1002.00 ^ab^ ± 172.5	978.97 ^a^ ± 53.5	1016.76 ^ab^ ± 118.3	1147.99 ^b^ ± 66.8	997.05 ^ab^ ± 172.7			
Protein [g]	Winter	36.67 ± 8.4	37.14 ± 7.7	38.54 ± 13.4	39.86 ± 8.1	41.24 ± 7.1	ns	ns	ns
	Spring	36.41 ± 9.8	34.33 ± 4.8	40.59 ± 9.7	40.63 ± 8.3	41.50 ± 10.9			
Fat [g]	Winter	25.35 ± 8.5	25.15 ± 5.2	23.36 ± 6.2	28.74 ± 9.1	31.81 ± 6.9	ns	ns	ns
	Spring	25.06 ± 4.5	26.98 ± 5.1	24.14 ± 6.4	28.45 ± 8.4	26.18 ± 9.7			
Carbohydrates [g]	Winter	172.28 ± 24.8	174.23 ± 38.0	165.70 ± 26.8	194.21 ± 27.8	170.43 ± 16.6	ns	ns	ns
	Spring	165.66 ^ab^ ± 31.1	156.72 ± 14.5	166.13 ± 13.9	189.28 ± 19.4	155.54 ± 27.69			
Sodium [mg]	Winter	2113.87 ± 684.4	1755.03 ± 346.1	2096.78 ± 586.3	2117.7 ± 441.6	2121.5 ± 487.9	ns	ns	ns
	Spring	1783.96 ± 386.69	1622.01 ± 521.9	1918.75 ± 445.9	2096.57 ± 518.9	1959.64 ± 661.9			
Potassium [mg]	Winter	1967.86 ± 354.1	1751.15 ± 285.8	1825.04 ± 301.1	2203.17 ± 410.2	2054.52 ± 556.6	ns	ns	ns
	Spring	2203.14 ± 454.8	1736.21 ± 307.3	1923.27 ± 307.3	2136.21 ± 245.0	1729.14 ± 396.8			
Calcium [mg]	Winter	309.14 ± 174.7	361.80 ± 128.4	367.27 ± 144.4	433.97 ± 114.5	450.12 ± 98.7	ns	ns	ns
	Spring	368.82 ± 150.0	414.75 ± 92.7	363.57 ± 145.2	505.93 ± 131.8	434.44 ± 260.6			
Phosphorus [mg]	Winter	595.00 ± 145.7	722.67 ± 189.7	632.06 ± 190.1	727.18 ± 163.1	696.43 ± 129.2	ns	ns	ns
	Spring	593.25 ± 183.80	661.38 ± 111.3	714.01 ± 111.7	738.31 ± 157.7	679.97 ± 208.6			
Calcium/Phosphorus	Winter	0.50 ± 0.17	0.51 ± 0.14	0.59 ± 0.18	0.60 ± 0.13	0.65 ± 0.11	ns	ns	ns
	Spring	0.61 ± 0.14	0.62 ± 0.07	0.51 ± 0.13	0.69 ± 0.11	0.62 ± 0.13			
Magnesium [mg]	Winter	178.40 ± 49.39	199.64 ± 52.19	152.90 ± 31.63	192.36 ± 39.41	184.68 ± 48.53	ns	ns	ns
	Spring	176.82 ± 52.75	177.17 ± 38.00	203.97 ± 78.44	188.08 ± 37.67	182.64 ± 50.60			
Iron [mg]	Winter	6.45 ± 1.03	6.46 ± 1.70	5.57 ± 1.14	7.81 ± 1.58	5.90 ± 0.96	ns	ns	ns
	Spring	6.36 ± 1.61	6.03 ± 1.32	6.33 ± 1.06	6.38 ± 1.03	6.32 ± 1.45			
Zinc [mg]	Winter	5.15 ± 1.81	5.40 ± 1.34	4.58 ± 1.24	5.48 ± 1.16	4.98 ± 0.84	ns	ns	ns
	Spring	4.78 ± 1.21	5.02 ± 0.88	5.22 ± 1.79	6.77 ± 1.73	4.91 ± 1.18			
Copper [mg]	Winter	0.85 ± 0.21	0.77 ± 0.15	0.68 ± 0.11	0.90 ± 0.18	0.75 ± 0.26	ns	ns	ns
	Spring	0.82 ± 0.24	0.79 ± 0.15	0.82 ± 0.25	0.87 ± 0.19	0.72 ± 0.21			
Manganese [mg]	Winter	2.52 ^a^ ± 0.55	3.03 ^a^ ± 0.66	1.90 ^b^ ± 0.43	3.58 ^c^ ± 0.89	2.74 ^a^ ± 0.58	***	ns	ns
	Spring	2.39 ± 0.54	2.62 ± 0.31	2.25 ± 0.79	2.83 ± 0.91	2.24 ± 0.83			
Vitamin B_1_ [mg]	Winter	0.68 ± 0.29	0.66 ± 0.24	0.54 ± 0.18	0.69 ± 0.22	0.66 ± 0.13	ns	ns	ns
	Spring	0.64 ± 0.19	0.56 ± 0.12	0.62 ± 0.28	0.69 ± 0.18	0.55 ± 0.17			
Vitamin B_2_ [mg]	Winter	0.82 ± 0.35	0.91 ± 0.17	0.90 ± 0.37	1.11 ± 0.34	0.93 ± 0.13	*	ns	ns
	Spring	0.85 ^ab^ ± 0.34	0.88 ^ab^ ± 0.20	0.87 ^ab^ ± 0.22	1.14 ^a^ ± 0.21	0.82 ^b^ ± 0.22			
Niacin [mg]	Winter	8.89 ^ab^ ± 3.07	6.47 ^a^ ± 1.56	6.08 ^a^ ± 1.83	9.73 ^b^ ± 3.76	9.87 ^b^ ± 3.69	**	ns	ns
	Spring	8.09 ^ab^ ± 1.97	5.55 ^a^ ± 1.53	8.45 ^b^ ± 3.06	9.07 ^b^ ± 2.71	6.57 ^ab^ ± 2.13			
Vitamin C [mg]	Winter	78.71 ^ab^ ± 21.16	47.62 ^a^ ± 21.65	61.45 ^a^ ± 31.88	73.21 ^ab^ ± 25.97	100.86 ^b^ ± 25.85	**	ns	ns
	Spring	91.08 ^ab^ ± 30.48	60.19 ^a^ ± 21.11	72.51 ^ab^ ± 27.00	105.17 ^b^ ± 72.78	91.90 ^ab^ ± 57.21			
Vitamin A [µg]	Winter	813.60 ± 331.51	758.96 ± 471.01	914.68 ± 548.43	1650.41 ± 877.42	1081.20 ± 592.62	ns	ns	ns
	Spring	1011.72 ± 370.74	613.99 ± 384.36	652.89 ± 193.76	1010.04 ± 180.27	893.34 ± 168.92			
Vitamin E [mg]	Winter	3.37 ^ab^ ± 0.64	4.80 ^b^ ± 1.51	2.87 ^a^ ± 0.67	4.86 ^b^ ± 1.44	4.57 ^b^ ± 2.89	**	ns	ns
	Spring	4.18 ^a^ ± 1.02	4.16 ^ab^ ± 1.54	3.58 ^a^ ± 1.39	4.98 ^b^ ± 1.54	3.94 ^ab^ ± 1.43			
Vitamin B_6_ [mg]	Winter	1.07 ± 0.31	1.02 ± 0.18	0.95 ± 0.31	1.09 ± 0.32	1.04 ± 0.41	ns	ns	ns
	Spring	1.13 ± 0.36	0.84 ± 0.28	1.10 ± 0.32	1.17 ± 0.31	0.94 ± 0.24			
Vitamin D [µg]	Winter	1.01 ± 0.96	1.02 ± 0.38	1.34 ± 0.69	0.95 ± 0.68	1.26 ± 0.88	ns	ns	ns
	Spring	0.66 ± 0.24	0.96 ± 0.66	1.01 ± 0.60	0.89 ± 0.61	0.84 ± 0.55			
Glucose [g]	Winter	9.02 ^A^ ± 2.97	5.31 ± 1.98	6.92 ± 1.45	7.98 ± 1.74	5.54 ± 2.94	***	ns	*
	Spring	12.98 ^Ba^ ± 4.63	5.41 ^b^ ± 1.77	6.80 ^b^ ± 2.56	8.02 ^b^ ± 3.41	4.27 ^b^ ± 1.86			
Sucrose [g]	Winter	39.21 ^ab^ ± 11.04	41.21 ^ab^ ± 10.38	44.31 ^a^ ± 14.46	49.11 ^a^ ± 8.60	30.49 ^b^ ± 14.60	***	ns	ns
	Spring	37.00 ^ab^ ± 8.67	43.55 ^a^ ± 11.46	46.42 ^a^ ± 14.28	42.75 ^a^ ± 10.23	29.46 ^b^ ± 19.16			
Fructose [g]	Winter	10.42 ^Aa^ ± 3.16	8.91 ^ab^ ± 1.84	9.51 ^ab^ ± 3.51	9.76 ^ab^ ± 2.33	6.55 ^b^ ± 2.74	***	ns	*
	Spring	14.83 ^Bb^ ± 4.92	7.45 ^ab^ ± 1.96	8.21 ^ab^ ± 4.78	10.26 ^b^ ± 4.77	5.66 ^a^ ± 2.05			
Lactose [g]	Winter	3.40 ^a^ ± 3.04	6.92 ^ab^ ± 2.91	6.30 ^ab^ ± 4.15	8.01 ^b^ ± 3.67	9.00 ^b^ ± 3.55	***	ns	ns
	Spring	6.01 ± 3.97	8.17 ± 2.09	6.76 ± 3.27	11.93 ± 3.70	6.70 ± 4.59			
Starch [g]	Winter	82.94 ± 18.39	87.27 ± 34.01	69.74 ± 21.83	83.46 ± 23.11	75.58 ± 19.05	ns	ns	ns
	Spring	67.11 ± 20.20	68.91 ± 14.44	69.23 ± 26.94	86.64 ± 10.71	75.16 ± 22.27			
Fiber [g]	Winter	16.33 ^ab^ ± 1.56	15.11 ^ab^ ± 3.65	14.99 ^ab^ ± 3.21	17.56 ^a^ ± 3.00	12.44 ^b^ ± 3.43	**	ns	ns
	Spring	18.12 ^a^ ± 3.04	14.13 ^b^ ± 2.56	13.87 ^b^ ± 5.23	13.89 ^b^ ± 2.34	13.08 ^b^ ± 3.96			
Cholesterol [mg]	Winter	111.56 ± 31.96	133.46 ± 87.45	154.66 ± 64.96	171.07 ± 98.65	149.24 ± 40.64	ns	ns	ns
	Spring	103.49 ± 31.70	155.34 ± 91.65	126.37 ± 67.21	118.23 ± 52.47	147.59 ± 97.23			
SFA [g]	Winter	11.20 ± 3.37	10.82 ± 3.00	11.50 ± 3.90	13.85 ± 4.27	14.22 ± 3.53	ns	ns	ns
	Spring	11.94 ± 2.27	12.63 ± 2.41	10.87 ± 5.37	13.69 ± 4.77	11.58 ± 3.08			
MUFA [g]	Winter	8.51 ± 2.99	7.63 ± 2.05	7.43 ± 2.24	9.09 ± 3.76	9.46 ± 2.36	ns	ns	ns
	Spring	8.88 ± 2.02	8.52 ± 2.07	8.06 ± 2.16	9.15 ± 2.97	8.97 ± 4.68			
PUFA [g]	Winter	3.24 ± 1.26	4.44 ± 1.35	2.29 ± 0.59	3.45 ± 0.67	4.12 ± 1.58	ns	ns	ns
	Spring	2.39 ± 0.25	3.58 ± 1.10	3.01 ± 1.41	3.36 ± 0.69	3.36 ± 1.67			
PUFA/SFA	Winter	0.30 ± 0.19	0.46 ± 0.28	0.20 ± 0.05	0.26 ± 0.07	0.33 ± 0.46	ns	ns	ns
	Spring	0.21 ± 0.04	0.29 ± 0.08	0.32 ± 0.19	0.27 ± 0.11	0.28 ± 0.08			
PUFA n-3 [g]	Winter	0.44 ± 0.13	0.40 ± 0.18	0.45 ± 0.16	0.56 ± 0.20	0.65 ± 0.47	**	ns	ns
	Spring	0.49 ^ab^ ± 0.12	0.42 ^a^ ± 0.18	0.44 ^ab^ ± 0.13	0.59 ^ab^ ± 0.22	0.69 ^b^ ± 0.39			
PUFA n-6 [g]	Winter	2.79 ± 1.36	4.03 ± 1.30	1.84 ± 0.49	2.89 ± 0.54	3.48 ± 2.24	*	ns	ns
	Spring	1.85 ± 0.30	3.16 ± 1.08	2.57 ± 1.39	2.77 ± 0.70	2.67 ± 1.32			
PUFA n-6/n-3	Winter	7.81 ^ab^ ± 7.85	11.40 ^a^ ± 3.23	4.28 ^b^ ± 1.29	5.47 ^b^ ± 1.55	5.26 ^b^ ± 2.27	**	ns	ns
	Spring	4.00 ± 1.24	8.35 ± 4.02	6.03 ± 3.35	5.31 ± 2.16	4.07 ± 1.07			
Salt [g]	Winter	5.29 ± 1.71	4.39 ± 0.87	5.24 ± 1.46	5.30 ± 1.10	5.31 ± 1.22	ns	ns	ns
	Spring	4.46 ± 1.48	4.06 ± 1.03	4.79 ± 1.11	5.25 ± 1.30	4.53 ± 1.44			

Significant effects: *** *p* < 0.001; ** 0.001 ≤ *p* < 0.01; * 0.01 ≤ *p* < 0.05; ns—not significant; K—kindergarten; S—season. a, b: means. Different letters in the same row indicate differences at *p* < 0.05 between kindergartens. A, B: means with different letters in the same column differ at *p* < 0.05 between seasons. SFA—saturated fatty acids; MUFA—monounsaturated fatty acids; PUFA—polyunsaturated fatty acids.

**Table 2:** Energy and nutrients in preschool meals constitute 75% of the Polish norms and recommendations [8] and the UL calculated by the EFSA [21].

				Norm Realization [%]				
Energy	Norm	75% of the Daily Requirement	Season	Kindergarten A	B	C	D	E
and Nutrients								
Energy [kcal]	1400 ^1^	1050 ^1^	Winter	98.27	99.17	94.83	110.46	105.50
			Spring	95.43	93.24	96.83	109.33	94.96
Protein [g]	21 ^2^	15.75 ^2^	Winter	232.81	235.80	244.71	253.07	261.83
			Spring	231.19	217.95	257.71	257.98	263.50
Fat [g]	47 ^3^	35.25 ^3^	Winter	71.92	71.35	66.28	81.55	90.24
			Spring	72.58	76.54	68.49	80.70	74.28
Carbohydrates [g]	227.5 ^3^	170.63 ^3^	Winter	100.97	102.11	97.11	113.82	99.89
			Spring	97.08	91.84	97.36	110.93	91.16
Sodium [mg]	1000 ^4^	750 ^4^	Winter	281.85	234.00	279.57	282.37	282.87
			Spring	237.86	216.27	255.86	279.54	261.28
Potassium [mg]	1100 ^4^	825 ^4^	Winter	238.52	212.26	221.22	267.05	249.03
			Spring	267.05	210.45	233.13	258.93	209.59
Calcium [mg]	1000 ^2^	750 ^2^	Winter	41.22	48.24	48.97	57.86	60.02
			Spring	49.18	55.30	48.48	67.46	57.92
Phosphorus [mg]	500 ^2^	375 ^2^	Winter	158.67	192.71	168.55	193.92	185.72
			Spring	158.20	176.37	190.40	196.88	181.33
Magnesium [mg]	130 ^2^/250 ^5^	97.5 ^2^/187.5 ^5^	Winter	182.98/95.2	204.76/106.5	156.81/81.54	197.30/102.6	189.41/98.5
			Spring	181.35/94.3	181.71/94.5	209.21/108.8	192.91/100.3	187.33/97.4
Iron [mg]	10.0 ^2^	7.5 ^2^	Winter	85.97	86.14	74.29	104.17	78.71
			Spring	84.84	80.36	84.38	85.04	84.28
Zinc [mg]	5.0 ^2^/10.0 ^5^	3.75 ^2^/7.5 ^5^	Winter	137.41/68.7	144.07/72.0	122.20/61.1	151.71/75.9	132.88/66.4
			Spring	127.68/63.8	133.98/67.0	139.23/69.6	146.12/73.1	130.81/65.4
Copper [mg]	0.4 ^2^/2.0 ^5^	0.3 ^2^/1.5 ^5^	Winter	282.24/56.5	258.11/51.6	225.20/45.0	301.32/60.3	250.11/50.0
			Spring	272.28/54.5	263.20/52.6	272.48/54.5	289.21/57.8	241.44/48.3
Manganese [mg]	1.5 ^4^	1.13 ^4^	Winter	223.06 ^a^	267.79 ^a^	168.26 ^b^	334.90 ^a^	242.69 ^a^
			Spring	211.62	231.98	199.10	250.05	199.00
Vitamin B_1_ [mg]	0.6 ^2^	0.45 ^2^	Winter	151.47	147.39	120.53	153.56	146.71
			Spring	141.81	124.43	137.95	154.09	122.41
Vitamin B_2_ [mg]	0.6 ^2^	0.45 ^2^	Winter	182.69	202.35	199.53	245.81	206.45
			Spring	188.46 ^ab^	194.64 ^ab^	193.22 ^ab^	252.54 ^b^	181.45 ^a^
Niacin [mg]	8.0 ^2^	6.0 ^2^	Winter	147.61 ^ab^	107.52 ^a^	100.94 ^a^	161.70 ^b^	163.97 ^b^
			Spring	134.31 ^ab^	92.14 ^a^	140.36 ^b^	150.70 ^b^	109.07 ^ab^
Vitamin C [mg]	50.0 ^2^	37.5 ^2^	Winter	209.89 ^ab^	126.99 ^a^	163.87 ^a^	195.24 ^ab^	268.94 ^b^
			Spring	242.88 ^ab^	160.50 ^a^	193.36 ^ab^	280.46 ^b^	245.06 ^ab^
Vitamin A [µg]	450 ^2^/1100 ^5^	337.5 ^2^/825 ^5^	Winter	241.07/98.6	224.88/92.0	271.02/110.8	489.01/200.1	320.36/131.1
			Spring	299.77/122.6	181.92/74.4	193.45/79.1	299.57/122.6	264.69/108.3
Vitamin E [mg]	6.0 ^4^	4.5 ^4^	Winter	74.90 ^ab^	106.63 ^b^	63.83 ^a^	107.91 ^b^	101.58 ^b^
			Spring	92.81 ^a^	92.34 ^ab^	79.63 ^a^	110.66 ^b^	87.55 ^ab^
Vitamin B_6_ [mg]	0.6 ^2^/7.0 ^5^	0.45 ^2^/5.25 ^5^	Winter	217.36/20.4	243.06/19.5	247.91/18.0	220.29/20.7	216.01/19.8
			Spring	240.46/21.5	249.83/16.0	199.99/20.9	260.81/22.4	208.82/17.9
Vitamin D [µg]	15.0 ^4^	11.25 ^4^	Winter	8.96	9.11	11.90	8.44	11.20
			Spring	5.91	8.53	8.97	7.95	7.44
Glucose [g]	* 10% energy value		Winter	34.35 ^A^	20.21	26.38	30.39	21.09
			Spring	49.43 ^Ba^	20.61 ^b^	25.92 ^b^	30.56 ^b^	16.28 ^b^
Sucrose [g]		26.25	Winter	149.37 ^ab^	157.00 ^ab^	168.79 ^b^	187.10 ^b^	116.16 ^a^
			Spring	140.95 ^ab^	165.92 ^a^	176.85 ^a^	162.86 ^a^	112.21 ^b^
Fructose [g]			Winter	39.71 ^Aa^	33.93 ^ab^	36.23 ^ab^	37.19 ^ab^	24.95 ^b^
			Spring	56.48 ^Ba^	28.37 ^ab^	31.28 ^ab^	39.11 ^a^	21.54 ^b^
Fiber [g]	14 ^4^	10.5 ^4^	Winter	155.48 ^ab^	143.90 ^ab^	142.84 ^ab^	167.24 ^a^	118.51 ^b^
			Spring	172.57 ^a^	134.56 ^b^	132.08 ^b^	132.31 ^b^	124.60 ^b^
Cholesterol [mg]	300	225	Winter	49.58	59.31	68.74	76.03	66.33
			Spring	45.99	69.04	56.17	52.55	65.59
SFA [g]	15.6	11.7	Winter	95.72	92.51	98.26	118.34	121.56
			Spring	102.09	107.92	92.88	116.97	98.96
PUFA n-3 [g]	1.02 ^4^	0.77 ^4^	Winter	57.17	51.80	58.11	73.18	83.91
			Spring	63.59 ^ab^	54.40 ^a^	57.77 ^ab^	76.14 ^ab^	89.35 ^b^
PUFA n-6 [g]	6.22 ^4^	4.67 ^4^	Winter	59.84 ^ab^	86.34 ^a^	39.35 ^b^	61.81 ^ab^	74.51 ^ab^
			Spring	39.61	67.77	54.93	59.31	57.27
Salt [g]	2.5	1.88	Winter	281.26	233.58	278.96	281.88	282.35
			Spring	237.39	215.90	254.65	279.02	241.18

* The 10% energy value of the food ratio. ^1^ Estimated Energy Requirement (EER); ^2^ Recommended Dietary Allowance (RDA); ^3^ Reference Intake ranges (RIs); ^4^ Adequate Intake (AI); ^5^ tolerable upper intake level (UL). SFA—saturated fatty acids; MUFA—monounsaturated fatty acids; PUFA—polyunsaturated fatty acids. a, b: means with different letters in the same row differ at *p* < 0.05 between kindergartens. A, B: means with different letters in the same column differ at *p* < 0.05 between seasons.

**Table 3:** Comparison of the recommendations of the planetary health diet with the composition of Polish preschool meals.

Product Groups	* MI (g/Day) for an Intake of 2500 kcal/Day	** MI (g/Day) for an Intake of 1050 kcal/Day	Macronutrient Intake (g/Day) in Preschool Meals									
			Winter					Spring				
			Kindergartens									
			A	B	C	D	E	A	B	C	D	E
**Whole grains**	**232.0**	**97.44 g**	**10.0**	**28.0**	**29.0**	**27.5**	**45.0**	**3.0**	**7.5**	**51.0**	**16.0**	**53.0**
Starchy vegetables (potatoes)	50.0	21.0	75.5	62.0	101.0	98.0	70.0	64.0	58.0	110.0	81.0	50.0
Vegetables	300.0	126.0	146.5	98.0	117.0	174.5	93.0	130.5	135.5	136.5	160.5	152.0
Fruits	200.0	84.0	162.5	105.0	137.5	84.5	105.0	195.5	123.5	126.0	122.0	86.5
Dairy foods	250.0	105.0	93.0	133.0	95.0	113.5	149.0	104.0	167.0	125.5	164.0	101.0
**Protein sources:**												
**Beef, lamb, and pork**	**14.0**	**5.9**	**46.0**	**38.5**	**38.0**	**44.0**	**42.0**	**38.5**	**26.5**	**39.0**	**47.5**	**34.0**
**Chicken and other poultry**	**29.0**	**12.2**	**17.0**	**7.0**	**16.5**	**14.0**	**22.0**	**7.0**	**10.0**	**17.0**	**8.5**	**14.0**
Eggs	13.0	5.5	16.0	27.0	22.0	28.0	25.0	11.0	36.0	10.0	22.0	27.0
Fish	28.0	11.8	10.0	8.0	8.0	9.5	11.0	9.0	10.0	11.0	5.0	11.0
Legumes	75.0	31.5	7.5	12.0	8.0	4.0	3.0	7.0	14.2	4.0	0.0	20.0
Nuts	50.0	21.0	1.0	4.0	0.0	0.0	4.0	0.0	0.0	3.5	0.0	0.0

* MI—macronutrient intake (grams per day) for an intake of 2500 kcal/day, in accordance with scientific targets for a planetary health diet (Willett et al., 2019) [13]. ** MI—macronutrient intake (grams per day) for an intake of 1050 kcal/day, equivalent to 75% of daily nutrition. Values calculated for preschool meals are based on the 2500 kcal/day planetary health diet recommendation (Willett et al., 2019) [13].

**Table 4:** Food Frequency Questionnaire results across the five preschools in the Upper North Shore area, Australia.

Food Group N	Foods	Consumption per Preschool Group During the 10-School-Day Study Period					The Form in Which the Food Was Consumed in the 10-Day Study Period				
		Kindergartens					Kindergartens				
		F	G	H	I	J	F	G	H	I	J
Group 1	Cereals, grains, roots, and tubers—rice, pasta, bread, potato, sweet potato, cassava, etc.	200	160	200	180	200	Bread Pasta Rice Spaghetti	Bread Pasta Rice Potatoes	Bread Pasta Rice Spaghetti	Bread Pasta Rice Potatoes	Bread Pasta Rice Spaghetti
		In total: 940 times (14.5%)									
Group 2	Pulses, legumes, and nuts—beans, lentils, soy, chickpea, peas, nuts, etc.	2	4	1	2	1	Chickpea hummus	Chickpea hummus Peas Beans	Peas	Beans Chickpea hummus	Lentils
		In total: 10 times (0.1%)									
Group 3	Milk and other dairy products—fresh milk, yogurt, cheese, sour cream, etc.	160	80	200	160	200	Fruit yogurt Cheddar cheese Milk Philadelphia	Fruit yogurt Cheddar cheese Philadelphia	Fruit yogurt Cheddar cheese Philadelphia	Fruit yogurt Cheddar cheese Philadelphia	Fruit yogurt Cheddar cheese Milk Philadelphia
		In total: 800 times (12.3%)									
Group 4	Meat, fish, and eggs—beef, chicken, lamb, pork, ham, bacon, fish, seafood, eggs, etc.	200	160	220	200	180	Ham Chicken Canned tuna Salami Eggs Mince pies Meatballs Bolognese	Ham Chicken Canned tuna Eggs Meatballs	Ham Chicken Canned tuna Salami Meatballs Bolognese Fish fingers	Ham Chicken Canned tuna Salami Eggs Mince pies	Ham Chicken Canned tuna Salami Eggs Meatballs Fish fingers Bolognese
		In total: 960 times (14.8%)									
Group 5	Vegetables and leaves—carrots, onions, tomatoes, capsicums, green beans, lettuce, spinach, broccoli, asparagus, cauliflower, etc.	100	220	140	120	80	Carrots Cucumber Cherry tomatoes Broccoli	Carrots Cucumber Broccoli Cherry tomatoes Capsicum Celery Lettuce	Carrots Cucumber Cherry tomatoes Celery	Carrots Cucumber Cherry tomatoes Green beans	Carrots Cucumber Cherry tomatoes Lettuce
		In total: 660 times (10.2%)									
Group 6	Fruits—bananas, apples, mangoes, strawberries, watermelon, oranges, melon, peaches, etc.	100	420	80	160	140	Banana Apple Watermelon Strawberry Melon Mandarin Orange	Banana Apple Watermelon Strawberry Melon Mandarin Kiwi Raspberry	Banana Apple Watermelon Strawberry Raspberry	Banana Apple Watermelon Strawberry Melon Orange	Banana Apple Watermelon Strawberry Raspberry Melon Mandarin
		In total: 900 times (13.9%)									
Group 7	Oil, fats, and butter—vegetable oil, sunflower oil, olive oil, butter, coconut oil, palm oil, margarine, etc.	80	40	80	100	80	Butter Margarine	Butter Margarine	Butter Margarine	Butter Margarine	Butter Margarine
		In total: 380 times (5.9%)									
Group 8	Sugar and sweets—sugar, jam, cakes, candies, pastries, sugary drinks, honey, chocolate, etc.	380	200	460	360	440	Muffins cakes, candies, pastries, chocolate bars, chocolate jellies, fruit juices, sugary drinks, Up&Go	Muffins, cakes, pastries, chocolate bars, jellies, fruit juices	Muffins, cakes, candies, pastries, chocolate bars, chocolate jellies, fruit juices, sugary drinks, Up&Go	Muffins, cakes, candies, pastries, chocolate bars, chocolate jellies, fruit juices, sugary drinks	Muffins, cakes, candies, pastries, chocolate bars, chocolate jellies fruit juices, sugary drinks, Up&Go

**Table 5:** Australian preschools’ food group consumption frequency comparison with the planetary health diet.

Food Groups		Kindergartens					Planetary Health Diet
		F	G	H	I	J	
1	Group 8: Sugar and sweets (28.3%)	>2 times a day	1 time a day	<2.5 times a day	>2 times a day	<2.5 times a day	Not recommended
2	Group 4: Meat fish and eggs (14.8%)	1 time a day	>1 time a day	<1 time a day	1 time a day	1 time a day	A very small amount recommended
3	Group 1: Cereals, grains, roots and tubers (14.5%)	1 time a day	>1 time a day	1 time a day	>1 time a day	1 time a day	A good amount of whole grains recommended, the rest in moderation
4	Group 6: Fruits (13.9%)	0.5 times a day	<2.5 times a day	0.5 times a day	>1 time a day	<0.5 times a day	Mainly recommended as half of the daily food intake
5	Group 3: Milk and other dairy products (12.3%)	>1 time a day	>0.5 times a day	1 time a day	>1 time a day	1 time a day	A very small amount recommended
6	Group 5: Vegetables and leaves (10.2%)	0.5 times a day	<1 time a day	<0.5 times a day	<0.5 times a day	>0.5 times a day	Mainly recommended as half of the daily food intake excluding starchy vegetables
7	Group 7: Oils, fats, and butter (5.9%)	>0.5 times a day	>0.2 times a day	>0.5 times a day	0.5 times a day	>0.5 times a day	Only a small amount of unsaturated plant oils recommended
8	Group 2: Pulses, legumes, and nuts (0.2%)	2 times every 10 days	4 times every 10 days	1 time every 10 days	2 times every 10 days	1 time every 10 days	A good amount recommended

## Data Availability

The original contributions presented in the study are included in the article, further inquiries can be directed to the corresponding author.

## References

[1] Tugault-Lafleur C.N., Black J.L., Barr S.I. (2017). A Systematic Review of Methods to Assess Children’s Diets in the School Context. Adv. Nutr..

[2] Myszkowska-Ryciak J., Harton A. (2018). Nutrition-related practices in kindergartens in the context of changes to legal regulations on foodstuffs used in canteen menus for children. Rocz. Panstw. Zakl. Hig..

[3] Jarman M., Edwards K., Blissett J. (2022). Influences on the dietary intakes of preschool children: A systematic scoping review. Int. J. Behav. Nutr. Phys. Act..

[4] Ronto R., Saberi G., Carins J., Papier K., Fox E. (2022). Exploring young Australians’ understanding of sustainable and healthy diets: A qualitative study. Public Health Nutr..

[5] Merkiel S., Chalcarz W. (2016). Preschool diets in children from Piła, Poland, require urgent intervention as implied by high risk of nutrient inadequacies. J. Health Popul. Nutr..

[6] Orkusz A. (2022). An Assessment of the Nutritional Value of the Preschool Food Rations for Children from the Wroclaw District, Poland—The Case of a Big City. Nutrients.

[7] Stroba M., Malinowska-Borowska J. (2021). Assessment of calcium and vitamin d content in meals served for preschool children attending to kindergartens in some Silesian cities. Rocz. Panstw. Zakl. Hig..

[8] Jarosz M., Rychlik E., Stoś K., Charzewska J. (2020). Nutrition Standards for the Population of Poland and Their Application.

[9] Verdonschot A., Follong B.M., Collins C.E., de Vet E., Haveman-Nies A., Bucher T. (2023). Effectiveness of school-based nutrition intervention components on fruit and vegetable intake and nutrition knowledge in children aged 4–12 years old: An umbrella review. Nutr. Rev..

[10] Wolnicka K., Taraszewska A., Jaczewska-Schuetz J., Korólczyk-Kowalczyk M. (2021). Nutrition in Kindergarten in Practice.

[11] Eat for Health Australian Dietary Guidelines 1–5. https://www.eatforhealth.gov.au/guidelines/australian-dietary-guidelines-1-5.

[12] Morgan E.H., Schoonees A., Sriram U., Faure M., Seguin-Fowler R.E. (2020). Caregiver involvement in interventions for improving children’s dietary intake and physical activity behaviors. Cochrane Database Syst. Rev..

[13] Willett W., Rockström J., Loken B., Springmann M., Lang T., Vermeulen S., Garnett T., Tilman D., DeClerck F., Wood A. (2019). Food in the Anthropocene: The EAT-Lancet Commission on healthy diets from sustainable food systems. Lancet.

[14] Vaidyanathan G. (2021). Healthy diets for people and the planet. Nature.

[15] Tulloch A.I.T., Borthwick F., Bogueva D., Eltholth M., Grech A., Edgar D., Boylan S., McNeill G. (2023). How the EAT–Lancet Commission on food in the Anthropocene influenced discourse and research on food systems: A systematic review covering the first 2 years post-publication. Lancet Global Health.

[16] Safe Sight Institute How a Vitamin A Deficiency Causes Vision Loss. https://www.sydney.edu.au/save-sight-institute/news-and-events/news/2020/how-a-vitamin-a-deficiency-causes-vision-loss.html.

[17] Wrzochal A., Gładyś-Jakubczyk A., Suliga E. (2019). Evaluation of diet in preschool-age children with Down syndrome–preliminary examination. Med. Stud..

[18] Myszkowska-Ryciak J., Harton A. (2018). Implementation of Dietary Reference Intake Standards in Preschool Menus in Poland. Nutrients.

[19] Searle B., Staton S., Littlewood R., Bayliss O., Thorpe K. (2024). A Missed Opportunity? Meal Provision in Early Childhood Education and Care Services in the Context of Socioeconomic Disadvantage. Matern. Child. Health J..

[20] (2018). Diet 6D, Computer Program.

[21] European Food Safety Authority (EFSA) Summary of Tolerable Upper Intake Levels, Version 4, September 2018, 1–4. https://www.efsa.europa.eu/sites/default/files/assets/UL_Summary_tables.pdf.

[22] (2022). NSW Education. https://education.nsw.gov.au/parents-and-carers/going-to-school/preparing/starting-preschool/food-at-preschool.

[23] (2013). Statistica.

[24] Thompson F.E., Subar A.F. (2017). Dietary Assessment Methodology. Nutrition in the Prevention and Treatment of Disease.

[25] Górska-Warsewicz H., Rejman K., Kaczorowska J., Laskowski W. (2021). Vegetables, Potatoes and Their Products as Sources of Energy and Nutrients to the Average Diet in Poland. Int. J. Environ. Res. Public Health.

[26] Beals K.A. (2019). Potatoes, Nutrition and Health. Am. J. Potato Res..

[27] ABS (2014). Australian Health Survey: Nutrition First Results: Food and Nutrients. https://www.abs.gov.au/statistics/health/health-conditions-and-risks/australian-health-survey-nutrition-first-results-foods-and-nutrients/latest-release.

[28] Orkusz A., Janczar-Smuga M., Frysiak D. (2018). Assessment of nutrition of children aged 4–6 years basing on decade menus. Zesz. Probl. Post. Nauk Roln..

[29] Monterrosa E.C., Frongillo E.A., Drewnowski A., de Pee S., Vandevijvere S. (2020). Sociocultural Influences on Food Choices and Implications for Sustainable Healthy Diets. Food Nutr. Bull..

[30] Sokoła-Wysoczańska E., Wysoczański T., Wagner J., Czyż K., Bodkowski R., Lochyński S., Patkowska-Sokoła B. (2018). Polyunsaturated Fatty Acids and Their Potential Therapeutic Role in Cardiovascular System Disorders—A Review. Nutrients.

[31] Gammone M.A., Riccioni G., Parrinello G., D’Orazio N. (2018). Omega-3 Polyunsaturated Fatty Acids: Benefits and Endpoints in Sport. Nutrients.

[32] ACER (2023). Developing Bright Minds from Birth to Age 12. https://www.acer.org/au/discover/article/developing-bright-minds-from-birth-to-age-12.

[33] Brown T.T., Jernigan T.L. (2012). Brain development during the preschool years. Neuropsychol. Rev..

[34] Simopoulos A.P. (2016). An Increase in the Omega-6/Omega-3 Fatty Acid Ratio Increases the Risk for Obesity. Nutrients.

[35] Czarniecka-Skubina E., Hamulka J., Jeruszka-Bielak M., Gutkowska K. (2024). Do Food and Meal Organization Systems in Polish Primary Schools Reflect Students’ Preferences and Healthy and Sustainable Dietary Guidelines? The Results of Qualitative Research for the Junior-Edu-Żywienie (JEŻ) Project. Foods.

[36] Manson A.C., Johnson B.J., Zarnowiecki D., Sutherland R., Golley R.K. (2021). The food and nutrient intake of 5- to 12-year-old Australian children during school hours: A secondary analysis of the 2011–2012 National Nutrition and Physical Activity Survey. Public Health Nutr..

[37] Garcia-Iborra M., Castanys-Munoz E., Oliveros E., Ramirez M. (2023). Optimal Protein Intake in Healthy Children and Adolescents: Evaluating Current Evidence. Nutrients.

[38] Nicklaus S. (2016). The role of food experiences during early childhood in food pleasure learning. Appetite.

[39] Orkusz A., Hapanowicz K. (2016). Assessment of the energy and nutritional values of meals in selected kindergarten in Wroclaw. Eng. Sci. Technol..

[40] Health Direct Australia Proteins. https://www.healthdirect.gov.au/protein.

[41] (2020). US Department of Agriculture and US Department of Health and Human Services Dietary Guidelines for Americans, 2020–2025. https://www.dietaryguidelines.gov/sites/default/files/2020-12/Dietary_Guidelines_for_Americans_2020-2025.pdf.

[42] Rizzoli R., Biver E., Brennan-Speranza T.C. (2021). Nutritional intake and bone health. Lancet Diabetes Endocrinol..

[43] Nekitsing C., Hetherington M.M., Blundell-Birtill P. (2018). Developing Healthy Food Preferences in Preschool Children Through Taste Exposure, Sensory Learning, and Nutrition Education. Curr. Obes. Rep..

[44] Bonjour J.P. (2011). Protein intake and bone health. Int. J. Vitam. Nutr. Res..

[45] Sartorius B., Sartorius K., Aldous C., Madiba T.E., Stefan C., Noakes T. (2016). Carbohydrate intake, obesity, metabolic syndrome and cancer risk? A two-part systematic review and meta-analysis protocol to estimate attributability. BMJ Open.

[46] Delli Bovi A.P., Di Michele L., Laino G., Vajro P. (2017). Obesity and Obesity Related Diseases, Sugar Consumption and Bad Oral Health: A Fatal Epidemic Mixtures: The Pediatric and Odontologist Point of View. Transl. Med. UniSa.

[47] Bogueva D., Marinova D., Raphaely T. (2017). Reducing meat consumption: The case for social marketing. Asia Pac. J. Mark. Logist..

[48] Institute of Medicine (2011). Dietary Reference Intakes for Calcium and Vitamin D.

[49] Shah S.C., Dai Q., Zhu X., Peek Jr R.M., Smalley W., Roumie C., Shrubsole M.J. (2020). Associations between calcium and magnesium intake and the risk of incident gastric cancer: A prospective cohort analysis of the National Institutes of Health-American Association of Retired Persons (NIH-AARP) Diet and Health Study. Int. J. Cancer.

[50] Health Council of the Netherlands (2018). An Evaluation of the EFSA’s Dietary Reference Values (DRVs), Part 1. Dietary Reference Values for Vitamins and Minerals for Adults, No. 2018/19A, Background document to: Voedingsnormen voor Vitamines en Mineralen voor Volwassenen No. 2018/19, The Hague. https://www.google.com/url?sa=t&source=web&rct=j&opi=89978449&url=https://www.gezondheidsraad.nl/binaries/gezondheidsraad/documenten/adviezen/2018/09/18/gezondheidsraad-herziet-voedingsnormen-voor-volwassenen/achtergronddocument%2BAn%2Bevaluation%2Bof%2Bthe%2BEFSA%2527s%2Bdietary%2Breference%2Bvalues%2B%2528DRVs%2529%2BPart%2B1.pdf&ved=2ahUKEwj9uZSS18aJAxUEslYBHUGWIgYQFnoECBUQAQ&usg=AOvVaw3Bsrpju4jTh-K5zap-8Oim.

[51] Healthline (2021). Is Salami Healthy? Nutrients, Benefits and Downsides. https://www.healthline.com/nutrition/is-salami-healthy.

[52] Domellöf M., Thorsdottir I., Thorstensen K. (2013). Health effects of different dietary iron intakes: A systematic literature review for the 5th Nordic Nutrition Recommendations. Food Nutr. Res..

[53] Li Z., Song X., Hang D. (2017). Dietary zinc and iron intake and risk of depression: A meta-analysis. Psychiatry Res..

[54] Li Z., Wang W., Xin X., Song X., Zhang D. (2018). Association of total zinc, iron, copper and selenium intakes with depression in the US adults. J. Affect. Disord..

[55] Kaganov B., Caroli M., Mazur A., Singhal A., Vania A. (2015). Suboptimal Micronutrient Intake among Children in Europe. Nutrients.

[56] Scientific Committee on Food (2001). Opinion of the Scientific Committee on Food on the Tolerable Upper Intake Level of Vitamin B1. https://food.ec.europa.eu/system/files/2020-12/sci-com_scf_out93_en.pdf.

[57] Scientific Committee on Food (2000). Opinion of the Scientific Committee on Food on the Tolerable Upper Intake Level of Vitamin B6. https://ec.europa.eu/food/fs/sc/scf/out80c_en.pdf.

[58] Kharrazian D. (2014). The Potential Roles of Bisphenol A (BPA) Pathogenesis in Autoimmunity. Autoimmune Dis..

[59] The President and Fellows of Harvard College The Nutrition Soutce. Vitamin C. https://www.hsph.harvard.edu/nutritionsource/vitamin-c/.

[60] Maggini S., Wenzlaff S., Hornig D. (2010). Essential role of vitamin C and zinc in child immunity and health. J. Int. Med. Res..

[61] Martini L., Pecoraro L., Salvottini C., Piacentini G., Atkinson R., Pietrobelli A. (2020). Appropriate and inappropriate vitamin supplementation in children. J. Nutr. Sci..

[62] Agostini D., Donati Zeppa S. (2023). Vitamin D, Diet and Musculoskeletal Health. Nutrients.

[63] Health Direct Vitamin D and Your Health. https://www.healthdirect.gov.au/vitamin-d-and-your-health#:~:text=The%20Australian%20government%20publishes%20recommended,of%20vitamin%20D%20per%20day.

[64] Horton-French K., Dunlop E., Lucas R.M., Pereira G., Black L.J. (2021). Prevalence and predictors of vitamin D deficiency in a nationally representative sample of Australian adolescents and young adults. Eur. J. Clin. Nutr..

[65] Aydoğan M., Korkmaz A., Barlas N., Kolankaya D. (2010). Pro-oxidant effect of vitamin C coadministration with bisphenol A, nonylphenol, and octylphenol on the reproductive tract of male rats. Drug Chem. Toxicol..

[66] Regulation of the Polish Minister of Health of 26 July 2016 on the Groups of Foodstuffs Intended for Sale to Children and Adolescents in Units of the Educational System and the Requirements to be Met by Foodstuffs Used as a Part of Collective Nutrition of Children and Adolescents in These Units (Dz.U. from 2016 poz. 1154). https://www.gov.pl/web/psse-bytom/rozporzadzenie-ministra-zdrowia-z-dnia-26-lipca-2016-r-w-sprawie-grupy-srodkow-spozywczych-przeznaczonych-do-sprzedazy-dzieciom-i-mlodziezy-w-jednostkach-systemu-oswiaty-oraz-wymagan-jakie-musza-spelnic-srodki-spozywcze-stosowane-w-ramach-zywienia-zbiorowego-dzieci-i-mlodziezy-w-tych-jednostkach-dzu20161154-z-dnia-20160801.

